# An intensive longitudinal dataset of in-game player behaviour and well-being in PowerWash Simulator

**DOI:** 10.1038/s41597-023-02530-3

**Published:** 2023-09-13

**Authors:** Matti Vuorre, Kristoffer Magnusson, Niklas Johannes, James Butlin, Andrew K. Przybylski

**Affiliations:** 1 Tilburg School of Social and Behavioral Sciences, Tilburg University, Tilburg, Netherlands; 2 Oxford Internet Institute, University of Oxford, Oxford, UK; 3 Centre for Psychiatry Research, Department of Clinical Neuroscience, Karolinska Institutet, & Stockholm Health Care Services, Region Stockholm, Stockholm, Sweden; 4 FuturLab Ltd, Hove, UK

**Keywords:** Human behaviour, Psychology

## Abstract

The potential impacts that video games might have on players’ well-being are under increased scrutiny but poorly understood empirically. Although extensively studied, a level of understanding required to address concerns and advise policy is lacking, at least partly because much of this science has relied on artificial settings and limited self-report data. We describe a large and detailed dataset that addresses these issues by pairing video game play behaviors and events with in-game well-being and motivation reports. 11,080 players (from 39 countries) of the first person PC game PowerWash Simulator volunteered for a research version of the game that logged their play across 10 in-game behaviors and events (e.g. task completion) and 21 variables (e.g. current position), and responses to 6 psychological survey instruments via in-game pop-ups. The data consists of 15,772,514 gameplay events, 726,316 survey item responses, and 21,202,667 additional gameplay status records, and spans 222 days. The data and codebook are publicly available with a permissive CC0 license.

## Background & Summary

Video games offer billions of players^1^, worldwide, opportunities for enjoyment, relaxation, competition, achievement, and socializing^2,3^. Although video game play has been studied for decades^4^, and despite widespread worries and hopes about games’ potential consequences^5,6^—and recent policy decisions to address those^7–9^—scientists are still uncertain about the psychological effects of play. Because many past studies have been limited by inaccurate self-reported play^10,11^, more recent efforts have focused on behavioral telemetry—data that are automatically collected by online game platforms and include player behaviors and in-game events^12–15^. While accurate, that telemetry, or what is made available of it, is typically aggregated and therefore doesn’t include potentially critical information about player behaviors and in-game events. As a consequence, while recent studies have been able to measure the quantity of play with great accuracy, the quality of play—what players do and what happens in the game—and its potential effects have remained unknown.

We describe a longitudinal study of video game play behavior, psychological well-being, and human motivations, and the resulting dataset^16^ that includes an unprecedented level of detail about 11,080 players’ unaggregated in-game play behaviors and events, in combination with their responses to psychological survey instruments, over 222 days of game play. We implemented a modified version of a commercially available game, PowerWash Simulator, that queried participating players’ psychological states during play using in-game popups. The combination of detailed play behavior and event data with players’ responses to psychological instruments within the game is suitable for both detailed descriptive studies and in-depth statistical modelling of video game play and its relations to players’ psychological states.

## Methods

**Fig. 1:**
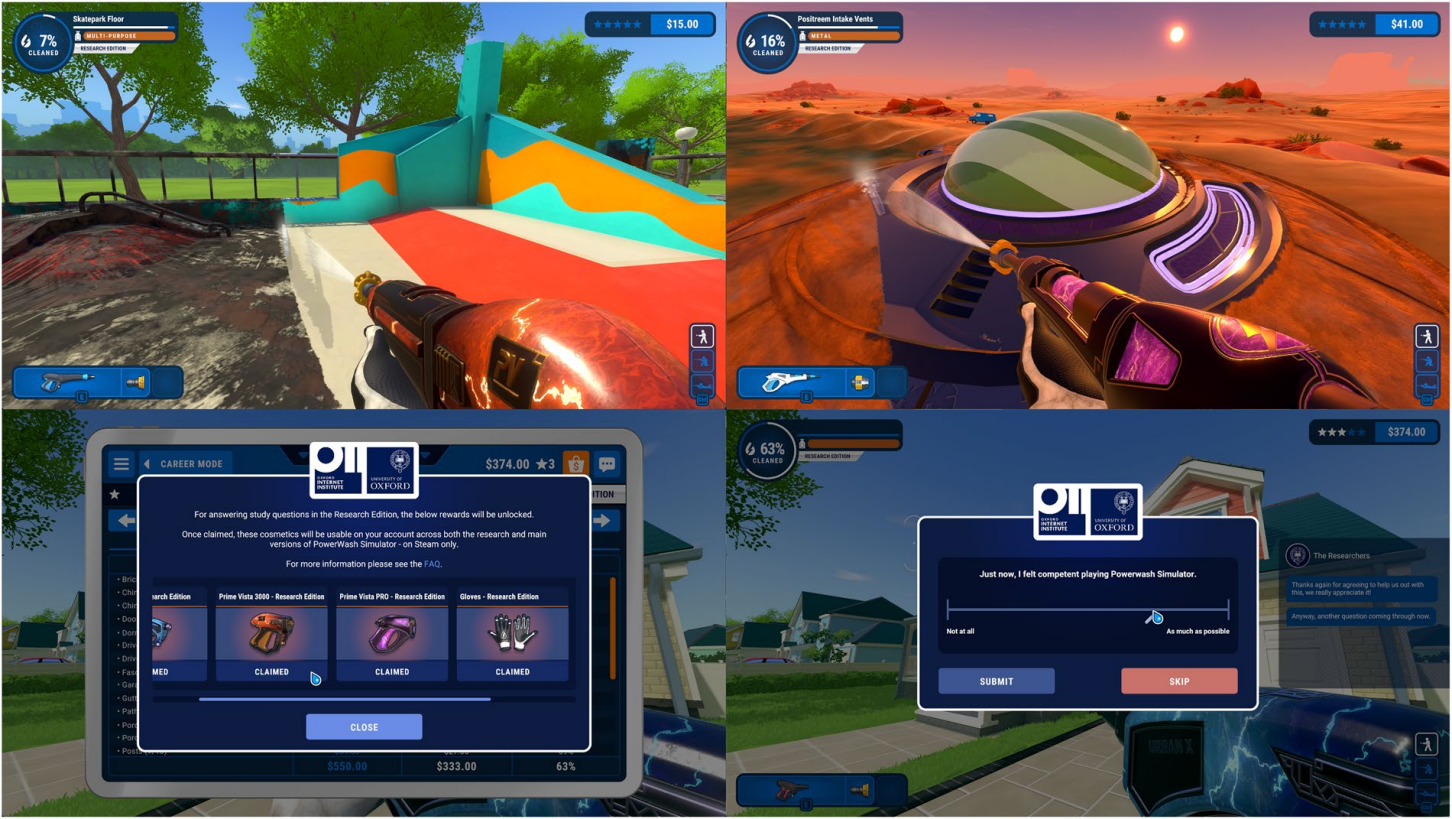
Screenshots of PowerWash Simulator gameplay. Top left: Powerwashing a skate park in the Playground level. Top right. Powerwashing a UFO in another level. Bottom left. Looking at the claimed rewards for participation in the study. Bottom right. Providing a Competence response using the in-game pop-up visual analog scale.

In this study, we developed a research edition of PowerWash Simulator (PWS)^17^, a first-person powerwasher game (see Fig. 1), and made it available on the same commercial platform (Steam; https://store.steampowered.com/) as the original game. Interested players, recruited via social media, word of mouth, and organic in-game links, downloaded the PWS research edition and gave informed consent to the study and data donation. We first describe the participants and the recruitment process, then the game and the modifications in the research edition, and then the psychological survey instruments that we used.

### Participant recruitment

The main PWS game is available on the Steam platform (at £19.99 on 2022-11-16). The study was first advertised on social media in August of 2022. Both FuturLab and Oxford Internet Institute also hosted websites that advertised the study and instructed potential participants on how to sign up to the study^18,19^. The launch of this study was also noted in news media^20^, which may have driven participation.

To participate, players were asked to use Steam to select the publicly available research edition of PWS, and then download it. After their first log-in, but before entering the game menu, participants read our consent form, indicated that they were 18 years or older, and agreed to voluntarily participate in the study. They then optionally answered basic demographic questions and proceeded to play as usual, but with the differences in the game design as discussed below. Eligible participants had to have purchased the game, and we did not pay for participation.

Then, in a second wave of recruitment in January 2023, the main branch of PWS was modified to include a button in the main menu that invited players to participate in the research branch. After participants joined the research branch of PWS, they were free to withdraw from the study at any point and return to the main branch of the study, and additionally request to have their data deleted.

### Participants

We defined a participant as anyone who provided informed consent, was over 18 years old, did not request their data to be deleted, and answered at least one survey item. 11,080 players participated, with a median age of 27 years (10th and 90th percentile: 19 and 40). The gender breakdown of our sample was as follows: Male: 6086 (54.9%), Female: 3190 (28.8%), Non-binary: 862 (7.8%), Transgender: 389 (3.5%), Other: 355 (3.2%), Prefer not to say: 125 (1.1%), Missing: 62 (0.6%), Intersex: 11 (0.1%).

**Fig. 2:**
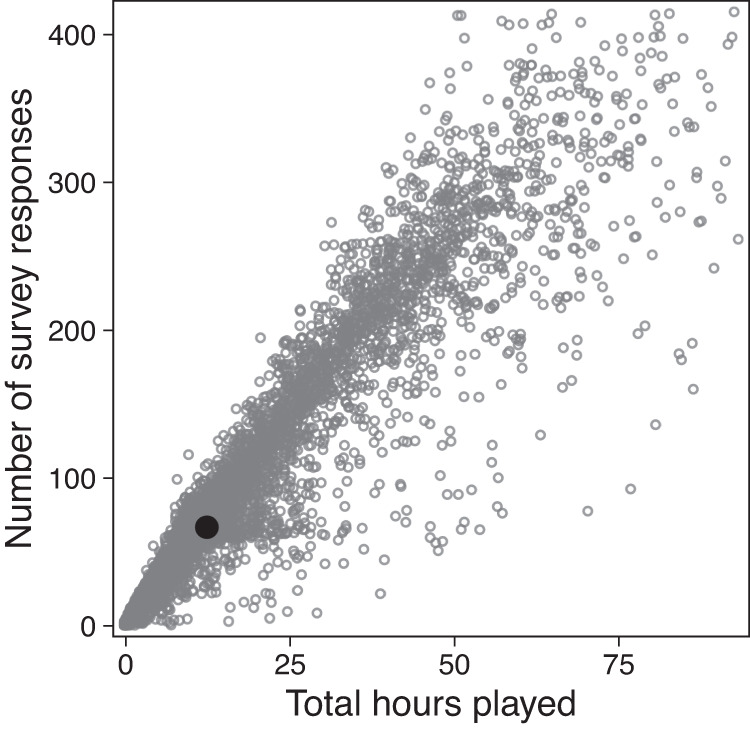
Summary of each participant’s contribution to the study. Both axes are trimmed at 99%. Filled dark point indicates the bivariate mean.

Because participants could withdraw from the research branch at any point, the duration of each player’s participation in the study, and the volume of data contributed, could vary. Figure 2 summarises each participant’s contribution to the study.

Although the main version of PWS is offered in a number of languages, the research version was only offered in English. Nevertheless, participants were located in 39 countries, with varying numbers of players from each country (USA (N = 5978), UK (N = 1074), Canada (N = 576), Germany (N = 531), Missing data (N = 410), Australia (N = 403), France (N = 292), Netherlands (N = 192), Japan (N = 160), Sweden (N = 125), South Korea (N = 122), Denmark (N = 91), Poland (N = 81), Belgium (N = 74), Brazil (N = 73), Finland (N = 72), Russia (N = 69), New Zealand (N = 64), China (N = 59), Singapore (N = 53), Norway (N = 47), Ireland (N = 44), Switzerland (N = 44), Austria (N = 43), Taiwan (N = 43), Czech Republic (N = 38), South Africa (N = 35), Argentina (N = 32), Spain (N = 32), Philippines (N = 30), Malaysia (N = 29), Italy (N = 25), Hungary (N = 23), Romania (N = 21), Portugal (N = 18), Ukraine (N = 18), Israel (N = 17), Mexico (N = 16), Iceland (N = 13), Thailand (N = 13)).

The study procedures were granted ethical approval by Oxford University’s Central University Research Ethics Committee (SSH_OII_CIA_21_011). All participants provided informed consent to participate and reported being 18 years or older.

### PowerWash simulator

In PWS, players control a character whose task is to use a power washer to clean various objects in different environments (Fig. 1; an illustrative video of the actual gameplay on the research edition of PWS is available on YouTube at https://www.youtube.com/watch?v=nIdOILxKsBA^21^). The main game offers a Career Mode, which is a sequential method of playing through the game’s levels in a curated order by cleaning all the dirty objects in the level, whilst accumulating more powerful washing equipment. Anecdotes and an underlying story are also delivered to the player via in-game ‘text messages’ at various progression intervals during Career Mode ‘jobs’. As the players progress, they earn credit which can be used to buy new washing equipment, soaps, and various cosmetic enhancements. In addition, upon completion, each level in Career Mode becomes available to replay at any time in Free Play. In Free Play jobs, players can use any equipment they have earned throughout their in-game career.

Before the game was released out of early access on Steam, we collaborated with PWS’s developer, FuturLab, to implement and make public a modified version of the main game. The academic researchers in our team created the conceptual design of the modifications to the main game, which JB then implemented. That research branch was identical to the main game, except for the following changes: First, it sent detailed game play data such as what happened in the game and when (see Data records, below) to the PlayFab service, from which we continuously exported data to an Amazon Web Services S3 storage bucket for intermediate storage.

Second, the research branch of the game was programmed to surface questions about the players’ psychological experiences and states during play using an in-game messaging and response system (Fig. 1D and Data records, below). Participants could also self-report their mood in the game menu (at most once every 30 minutes). Players’ responses to those prompts were sent directly to the academic researchers’ Qualtrics database, to which FuturLab members had no access. This ensured the privacy of the participants’ survey responses, even from the game developers.

Participating players received cosmetic items as a form of reward for taking part in the study branch. A cosmetic item was unlocked every 12 questions answered; there were in total 5 items to be unlocked. The rewards were usable in both the research and main versions of PWS. The rewards were not available otherwise in the main version of PWS.

The research edition also featured a “Research Edition” text in the game menu, to remind players that they are in the research version, and a button to return to the regular version. The research edition was only available in English, and excluded all downloadable content releases. Finally, although the main game had a multiplayer mode, we disabled that in the research version.

### Survey materials

The research version of PWS was programmed to surface psychological survey items to the player during game, and offered a menu item for self-reporting mood. These questions appeared as in-game pop-ups at most six times per hour of active game play, and with the constraint that they would be at least five minutes apart. Immediately before a survey question appeared, an in-game communication pop-up informed the participant that there would be a survey question soon (seen in the background of Fig. 1D). While the pop-up was visible, control of the player character was removed and a mouse cursor was made visible, and this was reverted after the participant’s response. All responses were optional and players could always click a “Skip” button. There were six different questions, but each pop-up included one item only, chosen at random from the six alternatives. Participants responded to all using a visual analog rating scale (VAS) with 1000 possible values. We show the questions and their associated samples sizes in Table 1.

**Table 1:** Counts of survey item responses.

Item	Observations	Players	Mdn(o/p)
Autonomy	72,529	5,871	22
Competence	71,975	5,858	21
Enjoyment	154,152	8,522	39
Focus	153,453	8,497	38
Immersion	72,298	5,853	22
Wellbeing	177,802	8,761	44
Wellbeing (menu)	24,107	5,851	9

*Note*. Mdn(o/p): Median observations per player. ‘Wellbeing (menu)’ indicates well-being responses that were volunteered through the game menu, instead of provided via the in-game prompts (see main text).

We queried subjective well-being with *“How are you feeling right now?”* with VAS endpoints *“Very bad”* and *“Very good”*^22^. Although feelings of well-being are often measured with separate positive and negative affect dimensions, our study required the least intrusive item possible to not disrupt play more than was necessary, and we therefore used the simple “happiness” question^22^. Furthermore, such single-item measures have been found to have good validity and are recommended in intensive longitudinal studies^23^.

In addition to well-being, we measured motivation and need satisfaction states with widely used items^24^. In order to capture the intrinsically motivating qualities of game play, we queried for “Enjoyment” with *“I was enjoying playing Powerwash Simulator just now.”* with VAS endpoints *“Not at all”* to *“As much as possible”*^13,24,25^.

We were also interested in the broad concept of being focused on playing the game, both in terms of a positively valenced absorption^26,27^, and a more “meditative” quality of attending rather than allowing one’s mind to wander^28,29^. We measured this quality of focusing on game play with the prompt *“I was totally focused on playing Powerwash Simulator just now.”* and a VAS from *“Not at all”* to *“As much as possible”*.

We also queried for players feelings of autonomy, the degree to which they felt autonomous in their decisions of how to play and what to do in the game, with *“Just now, I was doing the things I really wanted to in Powerwash Simulator”* with VAS endpoints *“Not at all”* and *“As much as possible”*. We queried their feelings of competence with *“Just now, I felt competent playing PowerWash Simulator”*, and feelings of immersion with *“Just now, I felt completely immersed in PowerWash Simulator”*, both with the same VAS as the autonomy item.

**Fig. 3:**
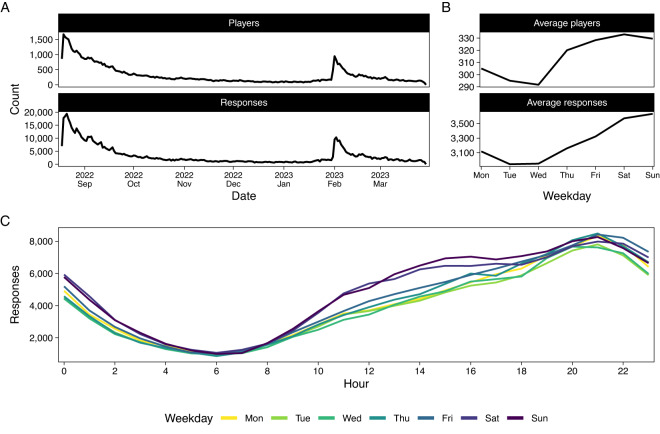
Survey response volumes over time. (**A**) Counts of daily survey responses and unique players responding to survey items over time. (**B**) Mean counts of survey item observations and unique players over the days of a week. (**C**) Total responses on each day of the week and each hour of the day. All numbers in A–C indicate counts of survey responses only, and not other game play behavior data.

The items were surfaced with equal probabilities, but the three latter motivation items were added to the study three weeks after launch. In addition, at every login, there was a 10% probability that the player was asked the well-being item. We summarize the volumes of participants’ responses to the survey items, including the in-menu self-reports, in Fig. 3.

### Game play data

**Fig. 4:**
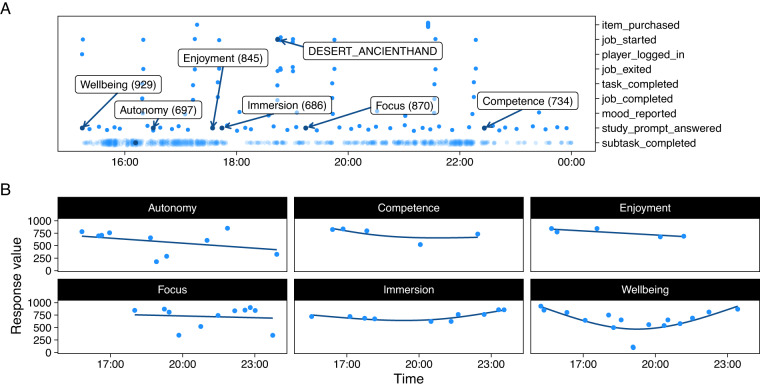
Figure of one player’s game play and survey data for one day. (**A**) Distribution of game play behaviors and events throughout the day. The y-axis indicates different events and behaviors, individual points are observations of those events when they happened (time; x-axis). We jittered the subtask_completed events vertically for visibility. The text labels show example variable values for each event (job_started shows the job name; subtask_completed shows the subtask name). (**B**) Survey responses over time. Lines are exploratory generalized additive model fits.

The research edition of PWS recorded 21 variables describing different facets of the player’s status when triggered by one of 10 in-game play behaviors (e.g. when the player completed a task) or 3 status updates (e.g. when the game was saved). For example, when the player completed a subtask (e.g. washed a window frame of the mansion; a “subtask_completed” *event*), the game captured and sent the current time (a *variable* with millisecond precision); the name of the subtask; the character’s current posture (standing, crouching, or prone); the character’s current coordinates in the level; which washer, nozzle, and extension the character was currently using; progression within the level (e.g. 80%); and the current game mode (e.g. career mode or free play). The events and their associated sample sizes are listed in Table 2. The spread of these events over a typical day of play are illustrated in Fig. 4. The events and variables are described in detail in the associated codebook^16^.

**Table 2:** Counts of game play events.

EventName	Observations	Players	Mdn(o/p)
exited_game	105,869	10,834	5
item_purchased	306,975	7,737	11
job_completed	155,100	10,105	9
job_exited	240,417	10,478	13
job_resumed	67,682	7,140	6
job_started	176,824	10,489	9
player_logged_in	113,004	11,080	5
subtask_completed	14,376,699	10,432	613
task_completed	212,818	10,107	17
study_reward_claimed	17,126	3,926	5
study_reward_unlocked	9,623	2,340	5
update_current_state	12,710,590	3,939	1,944
game_saved	8,482,454	7,725	456

*Note*. Mdn(o/p): Median observations per player.

However, not all variables were relevant to all events. Therefore, each event only triggered the saving of a subset of variables. In the above example, the “subtask_completed” *event* saved the values of 9 *variables*. The codebook explains which events were paired with which variables. We provide the dataset as a collection of tables, one for each event and only including the variables that were saved during that event. Therefore, for reconstructing more complex timeseries, users can fill relevant values forward or backward in time, wherever appropriate.

## Data Records

The data and codebook are openly available under a CC-0 license at 10.17605/OSF.IO/WPEH6 as a compressed .zip archive of event-specific comma-separated values (.csv) tables^16^. Those .csv files are exported from a DuckDB database^30^, and the repository associated with this manuscript shows examples of loading the tables to a DuckDB database for faster processing. Each row in these tables represents an event that occurred in the game, as listed in Table 2, including the survey response events (Table 1). The columns of those tables are variables of interest, which are further explained in the codebook. In addition, the demographics table includes basic person-level variables, such as the participant’s age, gender, country, and total number of survey responses contributed.

## Technical Validation

All data was automatically sent from the game running on the player’s device to either the PlayFab service or to the Qualtrics API. Therefore the likelihood of human error in data transport was minimized. Nevertheless, quality considerations remained due to possible technical problems in the internet connection between the player’s computer and PlayFab or Qualtrics, and due to improperly configured system clocks on players computers. We make our source code openly available for checking our assumptions regarding these data cleaning steps^16^.

## Usage Notes

The database is already cleaned and ready to be analysed with statistical software. Here, we provide a brief conceptual example on how the data can be used to answer substantive questions. A recent report^13^ found that there is a positive yet exceedingly small relation between time spent playing and well-being, such that people who on average play more tend to report greater well-being. However, that study and others like it have focused on longer time spans (typically two weeks) under which the estimated associations are assumed to operate. The current dataset, with psychological states and play measured practically in the same instance, allows examining associations operating at much shorter time spans.

**Fig. 5:**
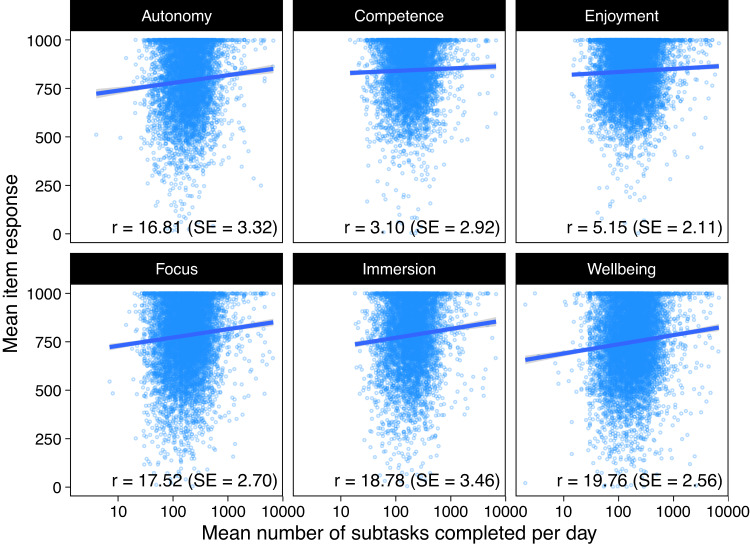
An example use of the database showing scatterplots of players’ mean item response and average game play engagement (mean subtasks completed per day). Points are individuals, the lines and numbers indicate regression slopes from a censored regression model of the outcome on the log mean number of subtasks completed. Note that the x-axis is on the log scale.

These data can be readily used to replicate that finding but under different assumptions about time spans in which the associations are assumed to operate. We loaded the “subtask_completed” and “study_prompt_answered” tables from the DuckDB database, aggregated the data to numbers of subtasks completed and mean response values for each individual. We then drew scatterplots and calculated regression slopes to illustrate the associations between average daily subtasks completed, as a proxy for game engagement, and each of the six psychological measures included in the study. The results conceptually replicated^13^; there was a positive correlation between well-being and game engagement (Fig. 5, “Wellbeing”). The code to conduct these analyses is included in the source repository of this manuscript^16^.

## Data Availability

The data used to clean the raw data is openly available at 10.17605/OSF.IO/WPEH6^16^. We processed the raw JSON files created by the game using jq and PostgreSQL. We then used R^31^ to post-process those files, including replacing the (already hashed) player IDs with new IDs to prevent reidentification of players by FuturLab Ltd.
